# TGF-β1 signalling in Alzheimer’s pathology and cytoskeletal reorganization: a specialized Tau perspective

**DOI:** 10.1186/s12974-023-02751-8

**Published:** 2023-03-13

**Authors:** Mahima Kapoor, Subashchandrabose Chinnathambi

**Affiliations:** 1 Neurobiology Group, Division of Biochemical Sciences, CSIR-National Chemical Laboratory (CSIR-NCL), Dr. Homi Bhabha Road, 411008 Pune, India; 2 Academy of Scientific and Innovative Research (AcSIR), Ghaziabad, 201002 India; 3 Department of Neurochemistry, National Institute of Mental Health and Neuro Sciences (NIMHANS), Institute of National Importance, Hosur Road, Bangalore, 560029 Karnataka India

**Keywords:** TGF-β, TGF-β1, Cytoskeleton reorganization, Extracellular Tau, Microglia, Alzheimer's disease

## Abstract

Microtubule-associated protein, Tau has been implicated in Alzheimer's disease for its detachment from microtubules and formation of insoluble intracellular aggregates within the neurons. Recent findings have suggested the expulsion of Tau seeds in the extracellular domain and their prion-like propagation between neurons. Transforming Growth Factor-β1 (TGF-β1) is a ubiquitously occurring cytokine reported to carry out immunomodulation and neuroprotection in the brain. TGF-β-mediated regulation occurs at the level of neuronal survival and differentiation, glial activation (astrocyte and microglia), amyloid production–distribution–clearance and neurofibrillary tangle formation, all of which contributes to Alzheimer's pathophysiology. Its role in the reorganization of cytoskeletal architecture and remodelling of extracellular matrix to facilitate cellular migration has been well-documented. Microglia are the resident immune sentinels of the brain responsible for surveying the local microenvironment, migrating towards the beacon of pertinent damage and phagocytosing the cellular debris or patho-protein deposits at the site of insult. Channelizing microglia to target extracellular Tau could be a good strategy to combat the prion-like transmission and seeding problem in Alzheimer's disease. The current review focuses on reaffirming the role of TGF-β1 signalling in Alzheimer’s pathology and cytoskeletal reorganization and considers utilizing the approach of TGF-β-triggered microglia-mediated targeting of extracellular patho-protein, Tau, as a possible potential strategy to combat Alzheimer's disease.

## Background

Alzheimer's disease (AD) is a progressive neurodegenerative disorder pathophysiologically characterized by extracellular amyloid-beta (Aβ) plaque, intracellular neurofibrillary tangles (NFTs) composed of hyperphosphorylated Tau protein, and neuronal death as well as synaptic loss leading to cognitive decline [1]. Neurodegeneration in Alzheimer's can be attributed to direct neurotoxic effects of the Aβ deposits or NFTs and indirectly to the chronic inflammation perpetuated by them. Brain resident cytokines can promote Aβ plaque and tangle formation, which in turn can over stimulate glial cells, majorly microglia and astrocytes, to produce these cytokines in excess. This potentiates a vicious positive feedback loop, making inflammation of the central nervous system (CNS) a significant clinical feature of AD pathogenesis [2, 3]. The amyloid cascade hypothesis centres on Aβ accumulation being the cardinal initiator of the cascade of events that culminate into neurodegeneration in Alzheimer's disease. According to this hypothesis, the aberrant cleavage of amyloid precursor protein (APP) carried out by β-secretase and followed by γ-secretase yields Aβ peptides, 38 to 43 amino acids in length, with Aβ_1–40_ and Aβ_1–42_ acknowledged as the most prevalent forms. Out of these, Aβ_1–42_ is the pathogenic, highly hydrophobic, more neurotoxic and aggregation-prone species incriminated in the molecular pathology of Alzheimer’s disease [4]. These Aβ_42_ peptide monomers can interact and group to give rise to Aβ oligomers (highest neurotoxicity), polymers, and, ultimately, highly stabilized, insoluble amyloid plaques [1, 5]. Treatment strategies to reduce the elevated Aβ load have failed to bring about complete cognitive recovery suggesting the amyloid cascade cannot solely account for various other pathological effects of AD pathogenesis. In addition, amyloid-targeting-based drugs or antibodies only address the consequential symptoms or a part of but not the overall pathology of AD. Simultaneously, multiple research findings at the time dictated that another protein Tau could be major player in AD pathology and progression. This led researchers worldwide to also contemplate Tau-based strategies in confronting the Alzheimer’s quandary.

Tau is a highly soluble, natively unfolded, hydrophilic protein encoded by the microtubule-associated protein tau (MAPT) gene on chromosome 17 [6]. It is classically known to regulate microtubule assembly, dynamics, organization and the microtubule-dependent axonal transport of biomolecules and organelles [7]. The association between mutations in the MAPT gene and development of fronto-temporal dementia with Parkinsonism-linked to chromosome 17 (FTLD-17) has paved the way for dysfunctional Tau, being considered sufficient for causing neurodegeneration and dementia even in absence of amyloid pathology. Tau-induced neurodegeneration can be attributed to its post hyperphosphorylation-linked detachment from microtubules and disruption of axonal transport. Hyperphosphorylated Tau can thus be self-assembled into paired helical filaments (PHF) or straight filaments (SF), leading up to neurofibrillary tangle formation: the final consequence of the Tau pathology process [7, 8]. Evidence gathered over the past decade suggests that the intermediate soluble oligomeric Tau species has a more central role to play in disease etiology [8]. Tau oligomers are small, diffusible complexes of two or more Tau molecules either phosphorylated or nonphosphorylated (mainly, 3 − 10 monomers), having β-sheet-rich structures that promote fibril formation beyond 20 nm size. Dimers have an apparent size of 94–130 kDa, while trimers range from about 150 − 190 kDa [9]. Their detection in the early Braak 0 and Braak 1 stages in brain tissue derived from AD patients has established their presence before NFTs detection [10]. They have been demonstrated to induce neuronal cell death and memory impairment even in an Aβ-aggregation-independent backdrop [9–12]. Studies also show that hyperphosphorylated Tau oligomers accumulated in the vicinity of the synapse mediate dysfunction of the ubiquitin–proteasome system and contribute towards synaptotoxicity in AD [13]. Impairment of fast axonal transport has also been attributed to pre-fibrillar Tau oligomers [14]. All of which point to Tau oligomers being responsible for early immediate Tau-mediated neurotoxicity in AD. Besides the traditional amyloid beta cascade and the microglial dysfunction–neuroinflammation hypothesis [15, 16], the concept of prion-like propagation of Aβ and Tau has emerged to bridge the knowledge gap in our understanding of the disease [1, 17].

## Prion model of transmission in Alzheimer's disease

Most neurodegenerative diseases-like Alzheimer's, with characteristic misfolded protein at their crux, tend to form aggregates that show prion-like seeding and propagation properties at molecular level, cellular level (within tissues), or between tissues (systemic level) as demonstrated by the wide range of studies [18–22]. Such misfolded proteins, often referred to as "propagons", behave as corruptive templates that interact with the normal proteins and bring about disease-causing conformational changes in them. These templates induce their aggregation, facilitate their elongation by recruiting monomers, promote their fragmentation and aid in their subsequent escape and intercellular transmission through neural networks [19, 23]. Thus, the model of prion-like propagation establishes the role of extracellular protein as an essential mediator of disease progression [2]. Amyloid fibril forming proteins like Aβ, Tau, α-synuclein, Hungtintin, and others demonstrate the unifying feature of β-sheets oriented perpendicularly to the long axis of the entire fibre, a structure popularized as "cross-β." These proteins can spread in a prion-like manner in the brain [19, 24].

Extracellular Tau species, in particular can be taken up into cells by employing diverse mechanisms, including macropinocytosis, micropinocytosis, bulk endocytosis, trans-synaptic pathway, heparan-sulfate proteoglycans, and tunnelling nanotube [17, 25]. Upon their entry, the Tau species in question (monomer/oligomer/aggregates) can actuate the aggregation of the intrinsic, cytosolic, non-aggregated Tau monomers in the recipient cell. Extracellular secretion of Tau, though vital in sustaining its trans-cellular movement, is poorly understood. One such model for Tau release is based on its pre-synaptic secretion-induced by action potential generated from α-amino-3-hydroxy-5-methyl-4-isoxazolepropionic acid (AMPA) receptor Ca^2+^ driven neuronal activity [26], while there are other models to suggest its non-synaptic secretion [17]. Recent work has implicated syntaxin 6 and syntaxin 8 of the exosome-based secretory pathway, particularly their transmembrane domain, in the extracellular release of cytosolic, aggregation-inclined proteins: α-synuclein and Tau in neurodegenerative disorders [27]. Findings have also held the transmembrane domain of Vesicle-associated membrane protein 8 (VAMP8), a vesicular SNARE protein located on late endosomes responsible for the excretion of Tau from neurons [28]. Moreover, it has been found that the low-density lipoprotein receptor-related protein 1 (LRP1) directly interacts with the lysine residues located in the MTBR region of Tau and regulates its uptake via endocytosis and its spread at cellular level [29].

Prion-like propagation of Tau oligomers has been evidentially demonstrated in varying degrees in the in vitro cellular assays in brain slices, cultured neurons and cultured microglia. In vivo demonstration has been carried out in post-mortem brains, brains of animal using brain homogenates extracted from Tauopathy patients or Tau transgenic mouse models, brain interstitial fluid samples and cerebrospinal fluid samples derived from mice models, conditioned media from cells transfected with Tau-aggregates or using recombinant Tau in the studies [17–19]. Braak stages can be used to define the distribution of phosphorylated Tau or neurofibrillary tangles (NFTs) propagated by Tau with respect to Alzheimer's progression. Furman et al. have used a FRET-based flow cytometry biosensor to check for evidence of seeding activity in freshly frozen human AD brain samples. They found that although the tangles are restricted to the entorhinal cortex area in the first stage, seeding activity of Tau was observed in the hippocampus and few neocortical areas. In stage 2 cases, Tau seeding was observed in frontal and parietal regions, but Tau pathology was only confined to the limbic regions [30]. Fewer instances of seeding in the cerebellum in stage 3 cases have been confirmed as NFTs do not generally localize in that region. They have proposed that early transcellular seeding of Tau may precede full-fledged development of Tau neuropathology [30]. In addition, microglial activation and Tau accumulation–propagation have been documented to occur in parallel with the progression of Braak stages in AD [31]. It has been demonstrated that soluble, high molecular-weight (HMW), phosphorylated Tau has the highest propensity for prion-like propagation between neurons [32]. Lasagna-Reeves and group injected Tau oligomers extracted from AD brain cortex into wild-type mice and observed the propagation of abnormal Tau conformation to normal endogenous murine Tau. They have thus confirmed that the Tau oligomer species is responsible for intercellular spreading [33]. Evidence for active transfer of Tau from cell-to-cell in the living human brain is still pending but studies performed by employing magnetic resonance imaging (MRI) and positron emission tomography (PET) point towards its fulfillment soon [6, 20].

## Microglial dysfunction and neuroinflammation in Alzheimer's disease

Microglia are the residential immune cells of the CNS originating from primitive myeloid precursors in the yolk sac and infiltrating the brain very early during embryonic development [34]. The neurotoxic and the neuro-regenerative effects of these cells, in relation to their phenotype, have been well-documented over the years. Microglia have two definitive functions: immune defence and maintenance of CNS homeostasis, which they accomplish by surveying the local microenvironment, migrating towards the beacon of pertinent damage and phagocytising cellular debris or pathological accumulates deposited in the brain [35, 36].

Fundamentally microglia have been simplistically classified into three states: M0 (Quiescent/ramified microglia): Under normal conditions, they are known to partake in homeostatic activity; their long extended, motile ramified processes aid in surveilling the CNS microenvironment for toxins, misfolded proteins, damaged neurons, injury, and invading pathogens. This state is also involved in routine phagocytic clearance of cellular debris. Microglia can show dual modes of activation: the M1 state corresponds to the classical mode of activation and confirms to pro-inflammatory, potentially cytotoxic proclivities of the microglia [36, 37]. This phenotype is known to secrete inflammation propagating cytokines such as TNF-γ, IL-1β, TNF-α, and IL-6 and reactive oxygen species (ROS) and nitric oxide (NO) post injury. The M2 state relates to an alternative mode of activation and confirms to the anti-inflammatory phenotype of microglia corresponding to tissue repair, extracellular matrix reconstruction and anti-inflammatory cytokine secretion [35, 37]. Though earlier devised to investigate the role of microglia in disease in a better way, mounting evidence has challenged the M1–M2 dichotomy doctrine and suggested that microglia activation occurs as a continuum with intersecting markers and not a fixed state; hence it cannot be subjugated to this overtly simplified model [38, 39]. Over the past two decades, studies have started looking into gene expression profiles rather than absolute M1/M2 phenotypes. Single-cell RNA sequencing (scRNA-seq) studies have analysed various heterogenous microglia clusters expressing disease-related genes and have revealed a subtype of human microglia linked specifically to Alzheimer’s [40]. Microglia coinciding with disease progression, i.e., stage-specific microglia particularly responsive to type I and type II interferon have also been recognised through scRNA-seq on AD mice models vs control [41].

A unique type of microglia, i.e., disease-associated microglia (DAM), marked by its own definite, distinctive pattern of gene expression has also been found via scRNA-seq. This pattern incorporates the decline in expression of homeostatic genes and increased expression of documented AD-linked genes, such as triggering receptor expressed on myeloid cells 2 (TREM2), apolipoprotein E (APOE) and TYRO protein tyrosine kinase-binding protein (TYROBP) [39]. Like the M1/M2 model, a study has also proffered classification of DAMs into pro-inflammatory and anti-inflammatory subtypes while addressing the fruitful prospects of targeting pro-inflammatory DAMs for therapeutics [42]. Ultimately regardless of the nomenclature in use, the profiling should coincide with establishing functionality in microglia. Recently transcriptomic studies in human microglia have revealed a Tau-specific microglial subset previously unidentified in mice [43, 44]. ScRNA-seq data have revealed an encompassing DAM signature that involves CX3CR1 and P2Y12R downregulation and upregulation of TREM2, SPP1, AXL, and APOE [44, 45]. Moreover, shared markers coinciding with DAM have been identified in a microglial subpopulation that shows upregulated TREM2 CD11c, APOE, LPL and CLEC7A, and downregulated TGFβ, TMEM119 P2RY12 and CSFR1 in aging and AD conditions [39, 44, 46]. Thus, transcriptomics/proteomic/metabolomic markers-based data in association with in-situ functional–spatial data should be taken into consideration for understanding dynamic microglial response to challenges in the CNS and their further application for therapeutics. Microglial surface receptors can recognize pathogens, cellular debris as well as misfolded proteins and induce a microglial response by getting activated. On activation, microglia can internalize these toxicity-inducing components by pinocytosis/phagocytosis/receptor-mediated endocytosis and can advance their degradation via endocytic cascades or through secretion of chemokines and interferons [47]. The immune stimulus usually subsides after the cell reverts to its normal physiological state, thus resolving its activated state. However, age-associated microglial dysregulation impairs its neuroprotective functions and makes it prone to sustained over-activation, thus contributing to further pathogenesis [47, 48]. Microglia exhibits a diverse range of phenotypes that show spatial and temporal changes in accordance to distance from the site of protein deposition and the exposure time-intensity of insult in the brain. This indicates their inherent potential to be modulated at various junctures along the trajectory of the AD to alleviate disease progression [47].

Elevated levels of pro-inflammatory cytokines in conjunction with increased microglial reactivity has been observed during advancement stages of a variety of diseases [49]. Moreover, activated reactive microglia have been found in abundance in the vicinity of NFT-associated neurons [50]. The amount of microglia activated in AD condition shows significantly higher correlation with NFTs as opposed to amyloid plaque distribution [51]. A study in P301S mutant human tau transgenic mice even disclosed that microgliosis (microglial reactivity) and synaptic dysfunction were observed months prior to NFT formation in Tauopathies [52]. Hyperphosphorylated soluble Tau can alter the normal phenotype of microglia thereon acting as a contributor of hampered surveillance of brain microenvironment, increased neuroinflammation, and neurofibrillary tangle formation [35, 53]. Moreover, co-localization studies undertaken by Nilson et al. point towards internalization and degradation of Tau oligomers by activated microglia, suggesting an intimate connection between progression of Tauopathies and underlying neuroinflammation [54]. It has also been revealed very recently via single cell RNA-sequencing studies that Tau-based stimulation can lead to an altered transcriptomic profile in primary microglia. This Tau-linked diseased state in microglia is exacerbated by NF-κB activation and inhibited by its inactivation [55].

Reactive microglia can internalize and phagocytose extracellular hyperphosphorylated Tau via a microglial receptor, the fractaline C–X3–C Motif Chemokine Receptor (CX3CR1) [56]. This receptor is integral for neuron–microglia crosstalk as its corresponding ligand, i.e., neuronal CX3CL1 binds to microglial CX3CR1 receptor, which keeps the proinflammatory-mediators released by microglia at bay [57]. A study by Bhaskar et.al. has also shown increased Tau hyperphosphorylation and aggregation as a direct consequence of LPS-mediated microglial activation. The same study employed human MAPT transgenic mice lacking CX3CR1 and revealed that CX3CR1 deletion augments hyperphosphorylation and aggregation of MAPT along with behavioural decline [58]. Bolos et al. further cemented the imperative role of CXCR1/CX3CL1 axis in phagocytosis of extracellular Tau and showed that its dysfunction contributes towards AD progression [56]. Tau also shows competitive binding to CX3CR1 receptor under diseased conditions. If the internalized Tau load is very high, it could have a detrimental effect on microglial phagocytic abilities leading to Tau secretion via the exosomal pathway [56, 59, 60].

Tau aggregates, oligomers, or fibrils activate microglia and upregulate the release of interleukin-6, a pro-inflammatory cytokine. Oxidative stress, a key triggering factor associated with formation of Tau tangles has been linked with reactive microglia and ROS release in AD [61]. Thus, a relationship of cyclical nature between Tau and the inflammation-directed pathways set in motion by microglia has come forth, wherein inflammation may pave the way for Tau-related pathophysiology, and once induced, Tau aggregates can reinforce and elevate neuroinflammation.

## TGF-β and Alzheimer's disease

A pleiotropic cytokine, Transforming growth factor-beta (TGF-β) is associated with neuroprotection, immunoregulation and modulation of growth–differentiation–survival at cellular level. It has three isoforms TGF-β1, TGF-β2, and TGF-β3, that are constitutively expressed in the central nervous system. TGF-β1, the most comprehensively studied isoform, functions as a neurotrophic factor involved in initiating and maintaining brain homeostasis, synaptic plasticity and neuronal differentiation. Its expression and release by astrocytes and microglia is considerably upregulated in the injured brain [62]. TGF-β receptor family comprises of the activin-like kinase 5 (ALK5) or TGF-β type I receptor (TβRI) subunit and the TGF-β type II receptor (TβRII) subunit with a serine–threonine kinase domain [63]. The binding of TGF-β ligand to TβRII enables recruitment of TβRI to form a heterotetrameric receptor complex, wherein TβRII receptor kinase transphosphorylates TβRI. As a result, the downstream components of the TGF-β canonical pathway, i.e., receptor-activated Smads: Smads 2 and Smad 3, are phosphorylated by TβRI [64]. This activated complex binds to Smad 4 and is further translocated into the nucleus to regulate target gene expression. TGF-β can also function via the Smad-independent, non-canonical pathways, such as Mitogen-Activated Protein Kinase (MAPK) pathway, the nuclear factor κB (NF-κB) pathway, and PI3K/Akt pathway [65]. The physiological role of TGF-β signalling in the brain is not clearly defined but is thought to span the arenas of CNS development, synaptic transmission, and neuroendocrine regulation [66]. Glial TGF-β1 might function as a instructive cue to neurons for transitioning from "growth state" to a "synaptogenic state" [66].

TGF-β-mediated regulation occurs at the level of neuronal survival and differentiation, glial activation (astrocyte and microglia), extracellular matrix production, amyloid production–distribution–clearance and neurofibrillary tangle formation, all of which contributes towards Alzheimer's pathophysiology [62, 67]. Various reports suggest that a decline in TGF-β1 signalling is closely linked with increased deposition Aβ and NFTs in Alzheimer's disease animal models [62]. Compared to healthy aged individuals, reduced levels of TGF-β1 [including active (25 kDa) as well as inactive (50 kDa) forms] were observed in plasma and serum of AD patients along with the decline in TGF-β1 released from peripheral blood cells in circulation [68, 69]. Patients with mild cognitive impairment have been found to be at a greater risk of developing AD due to reduced production of the anti-inflammatory TGF-β and increased production of proinflammatory TNFα [70]. Phosphorylated Smad2/3, unable to translocate to the nucleus in hippocampal neurons, is sequestered in the cytoplasm and closely associated with amyloid plaques and NFTs bringing about a decline in overall Smad-dependent TGF-β1 signaling [71]. Reduced phosphorylation of Smad2/3 noted in the AD affected brain is representative of improper functioning of TGF-β signaling [71]. Reduction in availability of nuclear Smad2/Smad3 and co-Smad4 occurs in the temporal cortical region in AD affected individuals’ brain [72, 73]. TGF-β1 signalling is relevant to the development of tau pathology as decline in transcription of TGF-β1 has been observed with an increment in NFTs in AD [74]. In AD conditions, impaired TGF-β/Smad signalling is the cause for dysfunctional Smad phosphorylation and its anomalous localization is contributory to hyperphosphorylation of Tau [3, 75].

In contrast, increased proportion of transforming growth factor-β1 in the CSF of Alzheimer's patients have been observed. Another conflicting study has shown increased TGF-β1 in the CNS of AD individuals [76, 77]. A positive correlation of this increase in TGF-β1 has been found with amyloid deposits accumulating in and perturbing vasculature of the brain thus accounting for cerebral amyloid angiopathy (CAA) observed in AD brains [77]. Consecutively, a correlated increase in serum levels of endoglin (a transmembrane glycoprotein on the endothelial cells) along with reduction in TGF-β1 has been associated with problems in cerebral microvasculature [78].

**Fig. 1:**
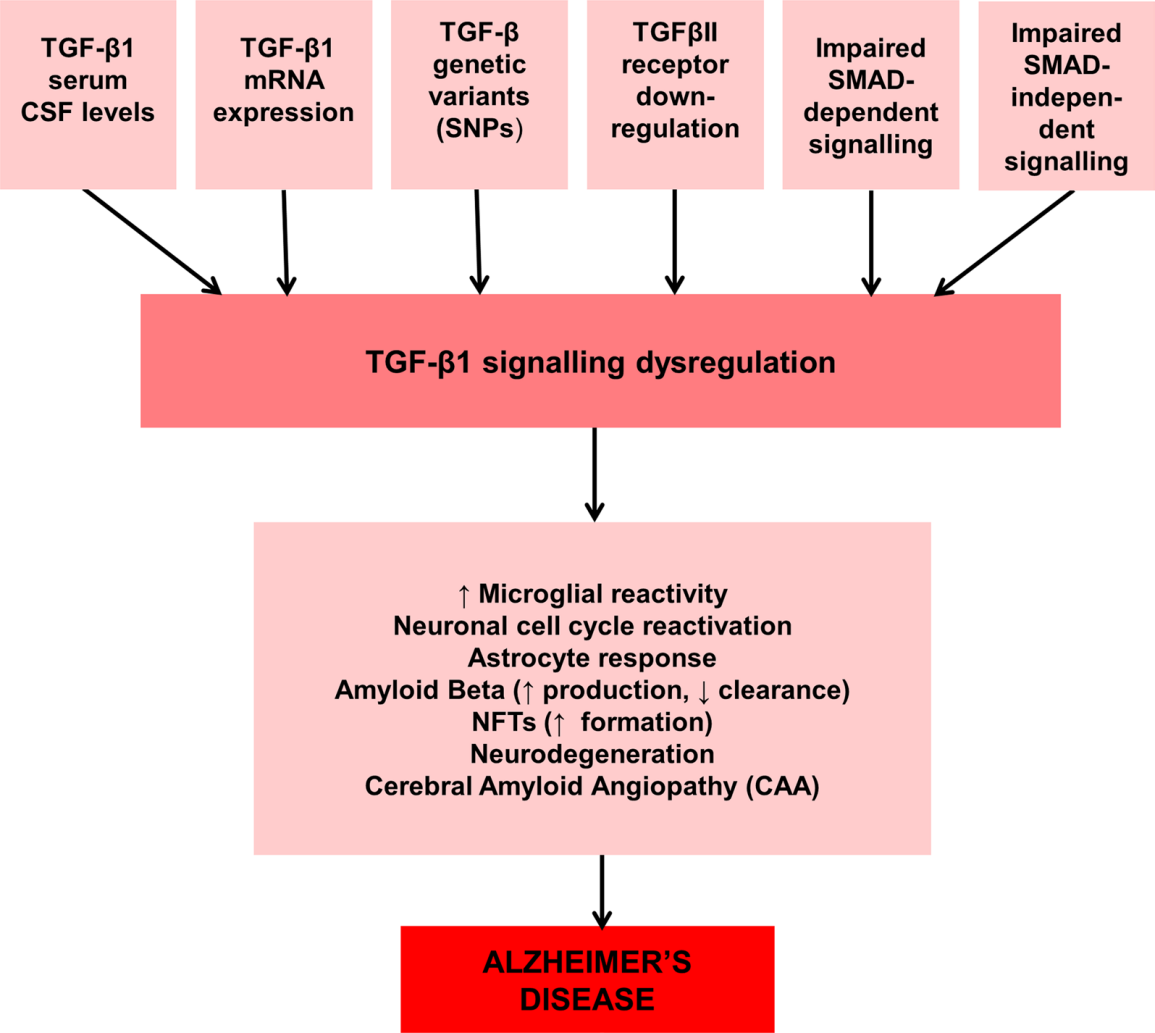
Impaired TGF-β1 signalling as a contributor to Alzheimer's Disease pathogenesis. Perturbations in TGF-β1 signalling, such as: (1) altered TGF-β1 levels in serum, plasma and CSF, (2) reduced expression of the TGF-β1 mRNA, (3) single nucleotide polymorphisms (SNPs) in TGF-β1 that in turn alter its expression, (4) downregulation of TGF-β1 type II receptor expression, (5a) dysfunction of SMAD dependent and (5b) SMAD independent signalling that results into a broad-spectrum of effects playing a contributory role in Alzheimer’s pathogenesis. These effects include increased microgliosis and astroglial reactivity, reactivation of neuronal cell cycle, increased formation and reduced clearance of Aβ and NFTs and occurrence of cerebral amyloid angiopathy which ultimately leads to full-fledged Alzheimer's disease

Certain genetic variations of transforming growth factor1 (TGF-β1), single nucleotide polymorphisms (SNPs) in particular have also been known to influence its expression, thereby presenting TGF-β1 as a prominent risk factor for Late-onset Alzheimer's disease (LOAD) [79]. Studies have put forward that TGF-β1 can reduce Aβ plaque deposits in the brain parenchyma, but it can alternatively increase Aβ incidence in cerebral blood vessels [80]. A trend of increased TGF-β levels with increasing age is observed in healthy aged individuals [81]. This can be correlated with a similar increase in AD incidence beyond 85 years and depletion in TGFβR2 in AD patient brains. Thus, a hypothesis has emerged through a perspective paper given by Fessel J., focusing on increasing TGF-β levels to counteract the problem caused by diminished TGFβR2 and to prevent the advancement of disease from mildcognitive impairment (MCI) into full-blown Alzheimer's [81, 82]. In its entirety, the literature suggests a crucial role of TGF-β1 signalling in AD etiology (Fig. 1).

## TGF-β and microglia

**Fig. 2:**
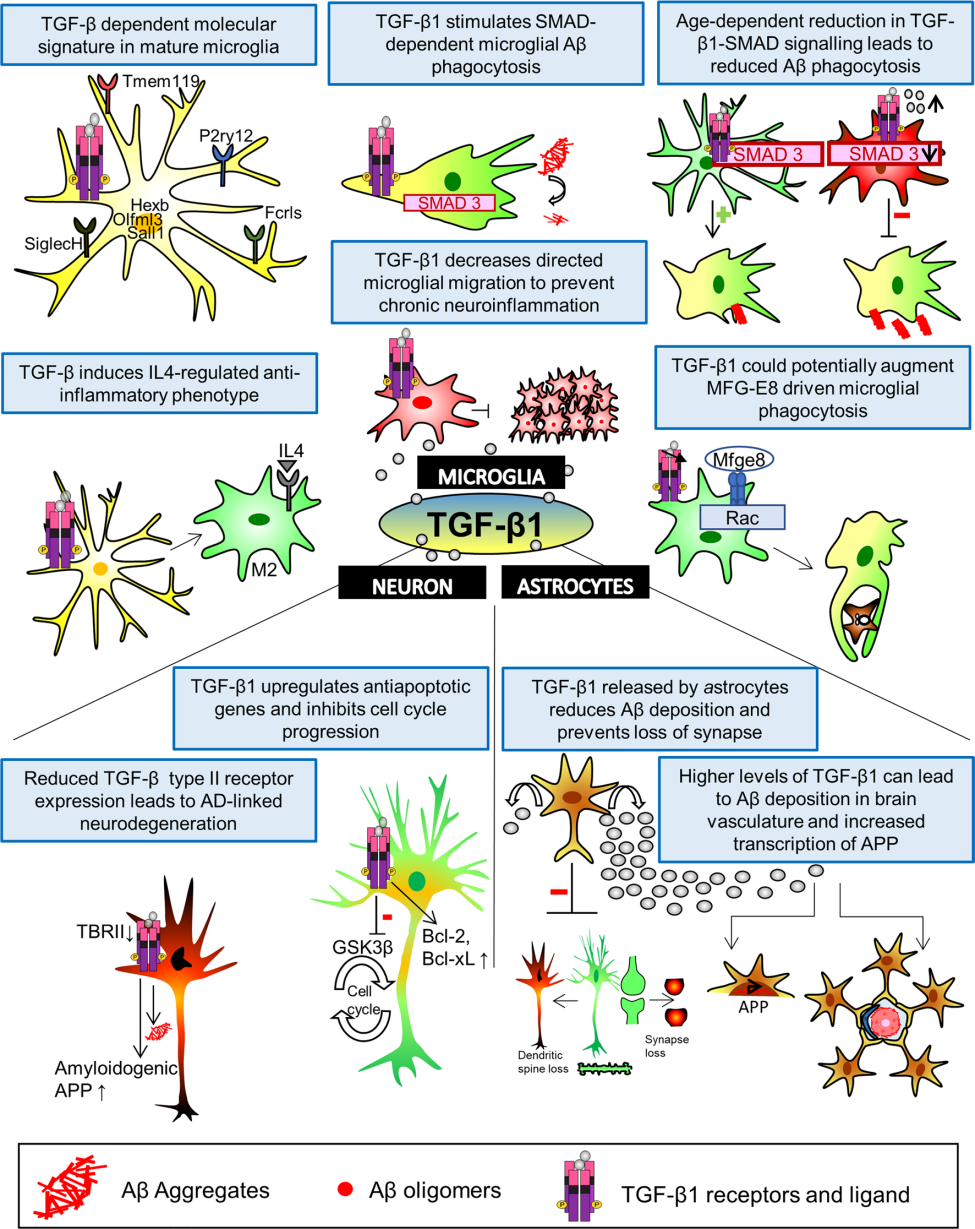
Effect of TGF-β1 on various cell types in the brain. *TGF-β and microglia*: TGFβ-dependent molecular signature specific to mature microglia has been found to induce expression of genes, such as P2rY12, Sall1, Fcrls, Tmem119, Olfml3, Hexb, and SiglecH. It also enhances IL4-induced alternative (anti-inflammatory phenotype) activation of microglia. Stimulatory effect of TGF-β1 helps in removal of Aβ by activating microglia-dependent phagocytic clearance via SMAD dependent signalling. Moreover, the TGF-β1-Smad3 signalling pathway shows age dependent effect on SMAD levels, which in turn leads to reduction in TGF-β1-induced Aβ phagocytosis in aged, adult microglia. MFG-E8 driven regulation of microglia-mediated phagocytosis has been well-documented. Recently, TGF-β was shown to increase MFGE8 production in myeloid-derived suppressor cells. *TGF-β and neuron:* TGF-β aids neuroprotection against Aβ-induced neurodegeneration via upregulation of the anti-apoptotic genes Bcl-xL and Bcl-2. In addition, TGF-β1 lead inhibition of cell-cycle and Wnt pathway rescue by activated PI3K-associated inhibition of the Tau-phosphorylating enzyme GSK-3β, is another mode through which it deploys neuroprotection against amyloid toxicity. Reduced TGF-β type II receptor (TβRII) expression in neurons and as the consequence reduced neuronal TGF-β signalling can also leads to AD-associated neurodegeneration. *TGF-β and astrocytes:* TGF-β1 produced by *a*strocytes reduces amyloid plaque deposition and prevents loss of synapse induced by AβOs. Increased release of TGF-β1 can lead to increased Aβ in brain vasculature and development of cerebral amyloid angiopathy. Furthermore, TGF-β1 contributes to increased transcription of Aβ precursor protein (APP) via Smad4, which might affect the overall Aβ deposition in AD brain

Owing to its clearly defined, essentially fundamental role as an anti-inflammatory mediator, TGF-β1 can endogenously regulate a plethora of microglial functions in the CNS. TGFβ1^−/−^ mutant mice have been known to exhibit increase in activated microglia proliferation and cortical neuron degeneration in neonatal stage suggesting it’s importance in maintaining neuronal health and microglial homeostasis [83]. TGF-β linked unique microglia-specific signature which, includes expression of Transmembrane protein 119 (Tmem119), Olfactomedin-like 3 (Olfml3), Purinergic Receptor P2Y12(P2ry12), Sal-like 1 (Sall1), Hexosaminidase Beta (Hexb), Fc receptor-like S (Fcrls), and Sialic acid-binding Ig-like lectin H (SiglecH) has been observed in mature microglia in homeostatic condition [84–88]. TGF-β1 can direct the extent of activation of microglia in the brain and facilitate parenchymal Aβ clearance by activated microglia [83]. Moreover, inductive effect of TGF-β1 has also been observed on microglia-mediated phagocytic clearance Aβ via SMAD dependent signalling [67, 89]. Suppression of TGF-β1 signalling has been found to contribute towards localised inflammatory environment arbitrated by macrophages and microglia under neurodegenerative conditions [3]. As opposed to that, TGF-β1 has also been demonstrated to cause a decrease in the Smad-linked, chemotactic, directed-migration of microglia towards Aβ aggregates. This also results in prevention of microglia-evoked neuroinflammatory responses [90]. Decrease in TGF-β levels in AD patients can favour the cytotoxicity brought by proinflammatory cytokines released by activated microglia and activate the expression of CD40/CD40 ligand complex by astrocytes and microglia [91]. Furthermore, depletion in SMAD3 levels have been observed in conjunction with aging-associated inflammatory environment. This affects the TGF–β1-Smad3 signalling pathway which in turn leads to reduction in TGF-β1-induced Aβ phagocytosis in aged, adult microglia [3, 92]. In addition to impaired Aβ phagocytosis, such aged microglia may undertake a senescent or dystrophic phenotype exhibiting attributes, such as the sustained release of cytokines abetting inflammation and high reactivity to oxidative stress [3]. However, contradictory evidence exists on aging-related change in microglial phagocytosis. Floden and Combs have observed decreased ability of microglia to phagocytose Aβ with age, while Von Bernhardi et al. have reported a slight escalation in the basal phagocytic ability of aged microglia, which cannot be stimulated by TGF-β or LPS activity [3, 93]**.** Milk fat globule-EGF factor 8 protein (MFG-E8) driven regulation of microglia-mediated phagocytosis has been well-documented [94, 95]. Recent studies have shown that TGF-β can acts as a promoter for increase in MFGE8 production in myeloid-derived suppressor cells thus suggesting that its regulation of microglial phagocytosis might involve MFG-E8 [96]. These studies all provide evidence that TGF-β1 plays an crucial role in microglial regulation (Fig. 2).

## TGF-β and neurons

TGF-β1 offers protection to neurons to counteract toxicity propagated by Aβ. This has been evidentiarily exhibited both in in vitro and in vivo AD models. Impairment of TGF-β signalling in neurons leads to Aβ deposition, overactivation of microglia, and neuronal degeneration [72]. TGF-β1 suppresses neuronal apoptosis by blocking the activation of caspase-3, sustaining the membrane potential of mitochondria and instigating anti-apoptotic gene expression (Bcl-2 and Bcl-xl) while inhibiting pro-apoptotic proteins via phosphorylation through ERK pathway in rat hippocampal neurons. It thus facilitates neuronal protection and survival against Aβ-induced neuronal death [72, 97–99]. Due to its role in maintaining post-mitotic neurons in their differentiated-anti-proliferative state, dysregulation in TGF-β1 signalling contributes to ectopic re-entry of neurons in the cell cycle induced by Aβ, consequently leading to problems with synaptic plasticity and cognition [2, 62, 68]. TGF-β1 also renders it’s neuroprotective effect by bringing forth PI3K-associated inhibition of GSK-3β, an enzyme known to increase phosphorylation of Tau, thus rescuing Wnt pathway and preventing hyperphosorylation of Tau and its disassembly from MTs (Caraci et al. 2008). A study carried out using group II metabotropic glutamate receptor agonists demonstrated the protective effect of release of glial TGF-β1 on cortical neurons against Aβ toxicity. Reduced neuronal TβRII expression in the initial stages of AD has been discerned in patients and mouse models [72]. As a direct consequence of that, reduced neuronal TGF-β signalling can leads to AD-associated neurodegeneration [2]. In addition, higher amount of Aβ deposits and β-secretase cleaved APP were observed in cultured neurons exhibiting reduction in TGF-β signalling. This suggests that decline in TGF-β signalling (due to reduced TβRII levels) contributes to increased Aβ deposition and neurodegeneration [2, 62]. TGF-β can also promote the clearance of amyloid β peptides in brain parenchyma through the activation of microglia, thus reducing neuronal loss [67]. The neuroprotective effect of TGF-β1 on neurons is summarized in Fig. 2.

## TGF-β and astrocytes

The effect TGF-β on astrocytes is immensely contextual, i.e., it can supress or elevate astrogliosis depending on the disease in question and the surrounding microenvironment [100]. Primary culture astrocytes when treated with TGF-β1 results in reactive astrogliosis [101], while the same cells exposed to proinflammatory mediators, such as IL-1α, TNF-α, and C1q when treated with TGF-β1, inhibit reactive astrogliosis [102, 103].

Categorically astrocytes have been classified as having the proinflammatory-A1 phenotype and neuroprotective-A2 phenotype similar to the M1/M2 classification of microglia [104]. However, the reactive states of astrocytes have been found to go beyond these two categories thereby making the scientific community abandon this binary gross division [104, 105]. However, astrocytes do acquire an inflammatory subtype with specialized markers that has significant relevance in pathological states. The distinct markers to identify and establish such neuroinflammatory astrocytes have been recently studied via scRNA-seq [105]. Such studies conducted in the human AD brain have revealed an astrocyte subpopulation with upregulated C3 complement in conjunction with amplified TGF β1 signalling [103, 106]

Overexpression of TGF-β1 is known to upregulate amyloid precursor protein (APP) expression in rodent astrocytes and human astrocytes, contributing to further Aβ accumulation by positively regulating APP promoter and carrying out stabilization of APP mRNA [107–110]. A remarkable observation along the same line is that TGF β1 regulation of APP expression is astrocyte-specific but does not extend to neurons. TGF β1 inhibition in APP mice astrocytes helps reduce Aβ production and has an overall beneficial effect analogous to its inhibition in ApoE knockout mice [103, 111]. Studies conducted in GFAP–TGF β1/APP transgenic mice have also shown that targeted overexpression of TGF β1 in astrocytes positively regulates Abeta40/42 production in them [103, 109]. Moreover, a study in transgenic mice model over-expressing the human APP with FAD mutations (Tg2576) documented surplus production of TGF-β around Aβ deposits in astroglia [110]. Another model over-expressing TGF-β1 in astrocytes shows the occurrence of vascular amyloid deposits in mice, thus underlining a correlation between increased TGF-β1 level and cerebral amyloid angiopathy in AD [77, 112]. Double transgenic mice overexpressing TGF-β1 and APP have been linked to faster Aβ deposition around blood vessels, possibly due to the stimulatory role of TGF-β1 on APP processing [77].

Contrary to that, TGF-β1 produced by astrocytes has indirect neuroprotective effects on other cell types, such as microglia or neuron. It can stimulate microglia to uptake Aβ thus preventing the decline in synaptic plasticity and memory impairment in Alzheimer's brain [67]. Fluoxetine, an antidepressant, increases astrocytic TGF-β1 secretion and deploys its neuroprotective action on AD models [113]. Astrocyte-derived TGF-β1 also protects hippocampal neurons from amyloid beta oligomer (AβO)-induced synapse loss, dendritic spine loss and memory loss [114]. Nominal increment in TGF-β1 released by astrocytes leads to a significant reduction of Aβ in the hippocampus of aged AD mice [67]. The effect of TGF-β1 on astrocytes is summarized in Fig. 2.

## Role of TGF-β in cytoskeleton remodelling and cell motility

Most motile cells can modulate or reorganize their cytoskeleton in response to external cues to facilitate their migration [115]. Microglia can also get activated, change shape to navigate restricted spaces in the brain parenchyma and migrate in response to signals from the external environment. The morphological changes to aid motility and mobility can be attributed to the rearrangement of actin filaments in particular.

**Fig. 3:**
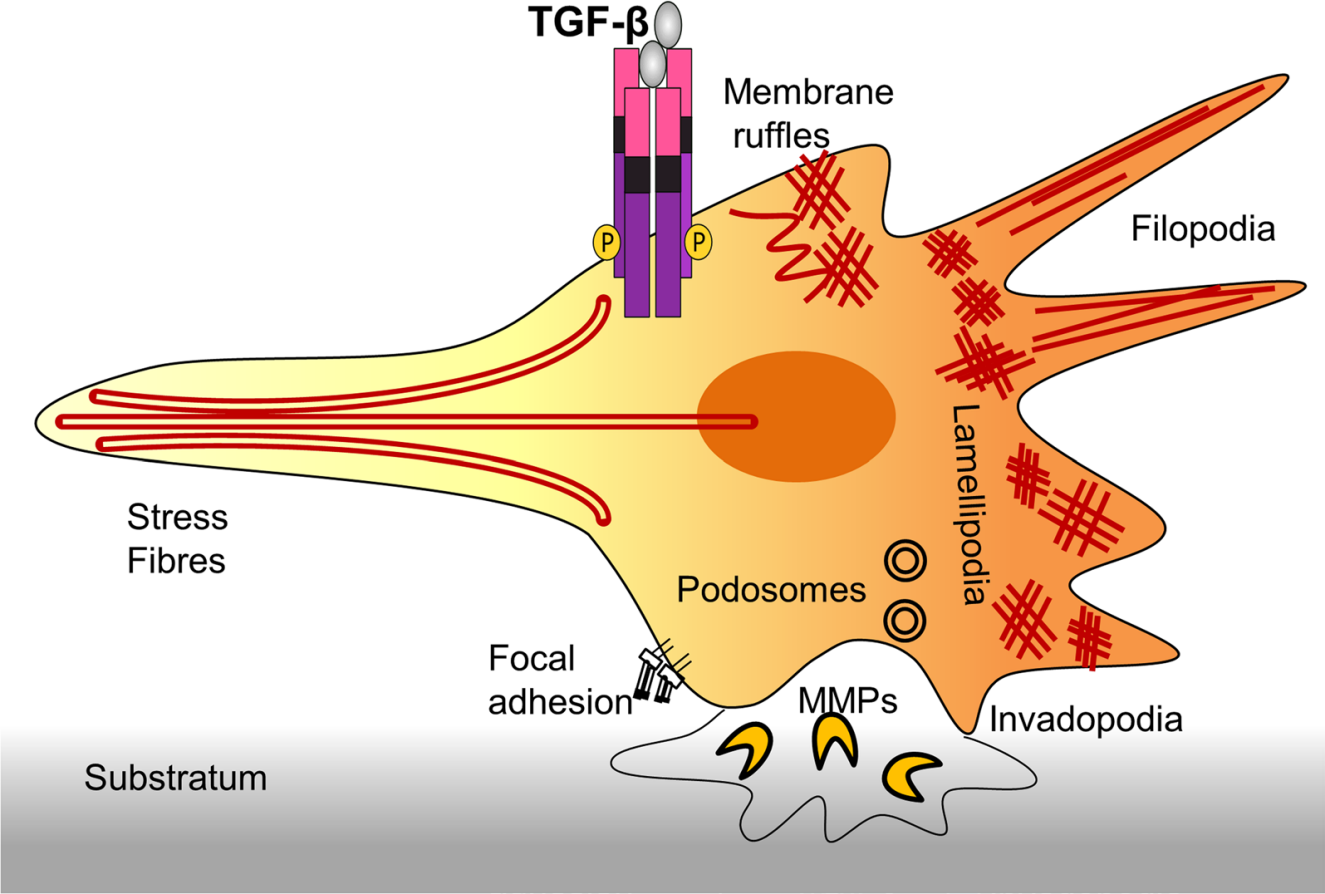
Types of cytoskeletal structures regulated by TGF-β in migratory cells. TGF-β can act as a migration inducer by enabling cytoskeleton rearrangements that result into formation of wide variety of migratory structures such as *membrane ruffles* are preliminary structures consisting of newly polymerized actin filaments found on the surface of a motile cell*; lamellipodia* thin sheet-like marginal membrane extensions composed of actin microspikes, found at the leading edge of a migratory cell. *Filopodia* go beyond the boundary of lamellipodia and occur as finger-like slender cytoplasmic projections-rich in parallelly arranged actin filaments; *podosomes* are dynamic structures with F-actin-rich core found on the ventral (outer) surface of the cell. They are rich in metalloprotein proteases (MMPs) that help in cellular propulsion by aiding extracellular matrix (ECM) degradation. *Invadopodia* are structures resembling podosomes found in cancerous cells. Like podosomes, they too help in cell invasion via proteolytic degradation of ECM; *focal adhesions* are integrin-containing large protein complexes occurring as point of contact between cell and ECM. They undergo cycles of assembly and disassembly as a migratory cell forms new contacts at the front end while detaching from the rear end; *stress fibres* constitute antiparallelly arranged actin filaments cross-linked by non-muscle myosin II. These structures provide necessary force for cellular contractility in non-muscle cells

The TGF-β superfamily of cytokines elicits a complex myriad of biological effects involving but not limited to cell–cell communication, cell–cell and cell–ECM adhesion, ECM remodelling and migration, proliferation, differentiation, regeneration, immune response, and apoptosis [115]. These effects differ in a context-specific manner based on the cell type, the receptor, and the specific ligand involved in signalling [115, 116]. It has been well-documented that TGF-β family members can influence the cytoskeletal architecture organization in cells. Overall, TGF-β can reorganise cellular cytoskeletal elements and remodel the extracellular matrix to accomplish differentiation and proliferation as well as to enable cell motility [63, 117]. Cytoskeleton reorganization triggered specifically in response to TGF-β signalling results into fast and short-term or durable and long-term modifications of actin dynamics which can alter morphological characteristics, adhesion ability, growth rate, motility and invasiveness of the cell [118]. Such dynamic rearrangements of actin-based structures brought about by TGF-β cues are capable of yielding structures such as: filopodia [118], lamellipodia [119], invadopodia [120], podosomes [121], and stress fibres [118] as well as inducing membrane ruffling [118] and focal adhesion remodulation [122] in context-dependent manner in specific cell types. Each of these structures instigate or support the migratory ability of microglia in one way or other. Membrane ruffles*,* formed of meshwork of newly polymerized actin filaments are observed at an early stage on the surface of motile microglia; Lamellipodia are found at the leading edge of a migratory cell [118]. They are composed of dense network of crosslinked actin filaments [119]. Filopodia go beyond the boundary of lamellipodia and help probe the cellular environment ahead of migrating cell. They occur as finger-like projections rich of parallelly arranged actin filaments [118]. Podosomes are dynamic adhesive structures present at the ventral surface of cells. They possess an F-actin rich core and an abundance of metalloprotein proteases (MMPs) that partake in forward propulsion of the cell by mediating extracellular matrix (ECM) degradation [121]. Invadopodia are podosomes-like structures found in cancerous cells. They too carry out proteolytic degradation of ECM and pay a crucial role in Epithelial-to-Mesenchymal Transition (EMT) during cancer [120]. Focal adhesions are integrin-containing large protein complexes occurring at the junction of cell and ECM as a link between intracellular actin filaments and extracellular substrate proteins. Their assembly occurs at the front end, while disassembly occurs as the cell detaches from the rear end to move forward [122]. Stress fibres are contractile actomyosin bundles providing protrusive force for mechanotransduction. They too undergo continuous cycles of assembly and disassembly [118]. Pictorial representation of the above-mentioned migration aiding structures formed as the result of effect of TGF-β on cytoskeletal remodelling can be found in Fig. 3.

## TGF-β-mediated SMAD-dependent cytoskeleton remodelling

TGF-β occurs as a latent complex with a prodomain (Latency-Associated Peptide—LAP) that, even on intracellular cleavage by furin following its production, remains noncovalently attached with the main C terminal, dimeric growth factor domain. This complex is secreted out in the extracellular matrix only on further binding with Latent TGF–β-Binding Protein (LTBP). Release of active TGF-β requires conformation change in the latent TGF-β1 complex brought about by mechanical and biochemical signalling mediated by binding of αV integrins to RGD domains on LAPs [123].

Active TGF-β ligands effectuate their cellular functions by first binding to type II TGF-β receptor, which then induces recruitment and formation of a heterotetrameric complex with type I receptor [64]. Phosphorylation of the TRI receptor by TRII in the glycine–serine-rich domain that lies adjacent to its kinase domain on the amino-terminal end leads to activation of its serine/threonine kinase activity. Activated TRI receptors can, thus, phosphorylate substrates and perpetuate signalling via both canonical SMAD2/3 dependent and non-canonical SMAD2/3 independent pathways [116, 124].

**Fig. 4:**
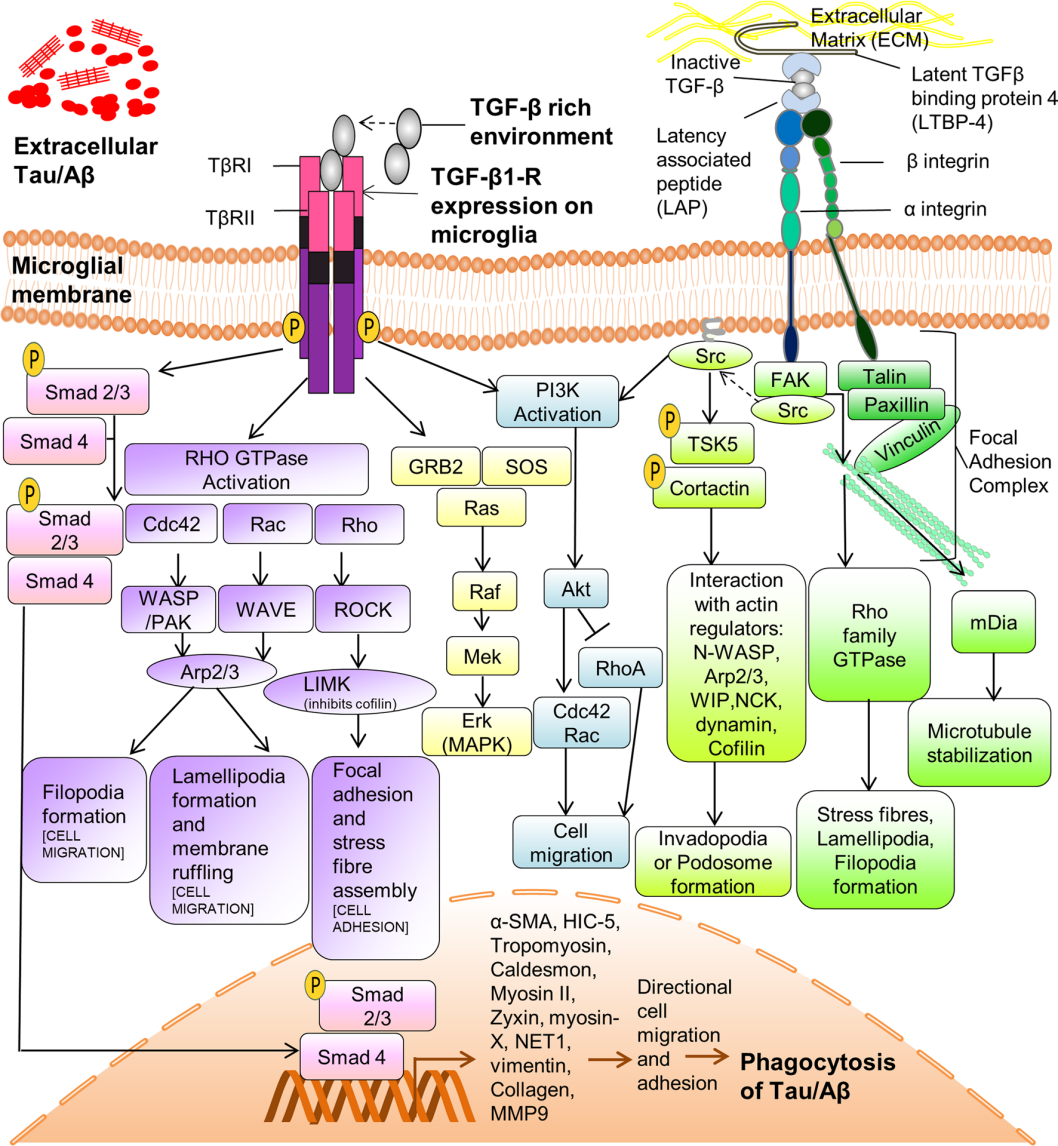
Signal transduction by TGF-β leads to cytoskeleton rearrangements through various pathways. TGFβ1 occurs as a latent complex associated with Latency-Associated Peptide (LAP) which on being secreted out further binds to the Latent TGF-β-Binding Protein (LTBP). Confirmation changes in this latent complex mediated by integrins is essential for the release of active TGF-β1. Active TGFβ1 ligand binds to TGFβ-RII receptor and which then phosphorylates TRI receptor thereby activating its kinase activity. This activated receptor can perpetuate TGF-β signalling via the canonical Smad-dependent as well as Smad-independent pathway. *Smad-dependent pathway: A*ctivated type I receptors directly phosphorylates Smad2/3 which then complex with a co-Smad, Smad 4.Translocation of the entire complex to the nucleus brings about transcription of various cytoskeleton linked genes such as α-SMA, HIC-5, Tropomyosin, Caldesmon, Myosin II, Zyxin, MT1-MMP, MMP9, Fibronectin, Collagen. *Smad-independent pathway:* TGF-β can activate Rho family GTPases [Rac, Cell Division Cycle 42 (cdc42), and Rho A] that act via fundamental actin–cytoskeleton reorganizers such as WASP/WAVE/ROCK and Arp2/3 leading to formation of migration inducing structures, such as filopodia, lamellipodia, stress fibres and focal adhesions. TGF-β can also regulate cell adhesion and migration via extracellular-regulated kinase(ERK) and phosphatidylinositol-3-kinase (PI3K) signalling. Furthermore, TGF-β operates through the Src kinase–Tks5 pathway to carryout ECM degradation with help of podosome and invadopodia. All of the following pathways converge onto membrane-associated actin remodelling that ultimately leads to cellular migration and subsequent pathoprotein (Tau) phagocytosis

Activated type I receptors can directly phosphorylate conserved transcription factors called receptor Smads (R-Smads), such as SMAD 1, 2, 3, 5, and 8. Most R-Smads post phosphorylation, particularly SMAD2/3 in mammals, complex with the Smad4 (a co-Smad), thus aiding nuclear translocation of the entire heterotrimeric complex from the cytoplasm, where it regulates transcription of an assemblage of genes [124, 125]. For SMAD dependent-signal transduction mediated by TGF-β leading to cytoskeleton remodulation refer (Fig. 4). TGF-β has been linked with modulating gene expression levels of numerous cytoskeleton-associated genes levels, such as myosin II, moesin, tropomyosin, caldesmon, and hydrogen peroxide inducible clone (HIC)-5, zyxin, myosinX, neuroepithelial cell transforming gene 1 (NET1) vimentin, fibronectin and collagen. Long-term actin cytoskeleton response mediated by TGF-β via its canonical SMAD pathway also results in transcriptional activation of RhoB and Alpha smooth muscle Actin (α-SMA) in fibroblasts [118, 126]. Hubchak et al. has implicated the Smad signalling pathway in expression of type I collagen in glomerular mesangial human cells. [127]. Besides migration, these genes even have ties to Epithelial–Mesenchymal Transition (EMT), a process necessary for embryonic development, wound healing, and cancer metastasis [128]. Various cytoskeletal genes regulated by TGF-β1 are summarized in Table 1.

**Table1:** Cytoskeleton-associated genes regulated by TGFβ1

Gene regulated by TGFβ	Function	Cell type	References
Tropomyosin and caldesmon	Tropomyosin: regulates actin filament stability, mediates the interaction between actin and myosin, and promotes stress fiber formation Caldesmon: binds to actin, myosin, tropomyosin, and Ca^2+^/calmodulin thereby regulating actin dynamics and actin–myosin contraction. Its effect on stress fiber formation can be both inhibitory or stabilizing depending on the cell type	Kidney epithelial cells, mammary epithelial cells	[128, 145–147]
Alpha-Smooth Muscle Actin	Mechanotransduction (increased contractility) in smooth muscle cells	In proximal tubular epithelial cells, TGF-β-mediated α SMA activation, depends on RhoA/Rho-associated protein kinase (ROCK), ERK1/2 p38MAPK and Smads	[148, 149]
Nonmuscle myosin II	Actin-based motor proteins that modulate cellular contractility, stress fiber formation, and migration	Epithelial cells	[150]
Hydrogen peroxide inducible clone (HIC)-5	It is a focal adhesion adapter protein partaking in the formation of stress fiber invadopodia, thus contributing to cell migration as well as matrix degradation	Various cell types TGF-β-induced HIC-5 upregulation is dependent on RhoA/ROCK signalling	[151, 152]
Zyxin	It occurs at the site of cell adhesion and interacts with the Ena/VASP family of actin-binding proteins to direct actin assembly/reorganization. It maintains the integrity of cell adherens junction and facilitates migration as well as invasion	Mammary epithelial cells, lung cancer cells	[153–155]
MyosinX	MyosinX promotes formation of parallel bundled F-actin filaments that is required for formation and elongation of filopodia	Fibroblast cells	[156, 157]
RhoB	RhoB regulates migration through change in focal adhesion dynamics–distribution and by stabilizing lamellipodial protrusions	Adenocarcinoma cell, primary endothelial cells, prostate cancer cells, breast cancer cells	[158]
NET1	It is a RhoA guanine nucleotide exchange factor (GEF) important for cell migration and invasion	Gastric adenocarcinoma	[85, 159]
Vimentin	Vimentin belongs to the intermediate filament family of proteins. It promotes cell migration by enabling contact-dependent cell stiffening. It can interact with and modulate the dynamics of microtubules as well as actin–myosin network	Fibroblasts, leukocytes, endothelial cells and astrocytes	[160, 161]

## TGF-β-mediated SMAD-independent cytoskeleton remodelling

TGF-β also induces morphological alterations and actin cytoskeleton reorganization in various cells through SMAD independent mechanisms. RhoA/ROCK signalling pathway acts as the fundamental mediator of cell movement. Short-term response to TGF-β exposure via Rho family GTPases [Cell Division Cycle 42 (cdc42), Rac and Ras-homolog gene family, member A (Rho A)] on activation of ROCK⁄LIMK⁄cofilin pathway leads to polymerization of actin [118, 126, 129]. Furthermore, TGF-β can help recruit fundamental actin–cytoskeleton reorganizers such as WASP/WAVE/ROCK and Arp2/3 through activation of Rho family GTPases, leading to formation of migration inducing structures-like filopodia (Cdc42), lamellipodia (Rac), stress fibres (Rho A) and focal adhesions (RhoA). RhoA can regulate actomyosin contractility and induce actin stress fiber formation via ROCK activation [130]. Inhibition of ROCK by its inhibitors has been reported to impede TGF-β-mediated stress fibre formation both in vitro and in vivo [128, 130]. Another Rho family member, Cdc42, is also a crucial regulator of cell polarity, motility, and contractility. Inhibition of ROCK/Cdc42-mediated microglial motility prevents increment in cell size, suppresses filopodia formation, and results in depletion of the microglial phagocyting domain [131, 132]. Meanwhile, prolonged TGF-β stimulus results in the formation of stable actin filament bundles (stress fibres) [118]. TGF-β signalling has been reported to also govern plasticity of the cell and its EMT transition that facilitates tumor progression as well as synchronised development of tissues/organs. Cytoskeletal remodelling is a characteristic feature of Epithelial–Mesenchymal Transition (EMT) and the genes regulating actin show an overshoot in expression during TGF-β-mediated EMT [128, 132].

Also, activated TβRI can directly phosphorylate ShcA, leading to the establishment of a ShcA–Grb2–Sos complex charged with the task of activating Ras localised on the plasma membrane. Activated Ras then enables sequential activation of Raf, MEK, and ERK (MAPK)[133]. TGF-β can also induce actin reorganization via extracellular-regulated kinase (ERK) signalling, thereby regulating cell adhesion and migration. Moreover, TGF-β1-mediated ERK–MAP kinase activation in mesangial cells further enhances expression of collagen and activation of Smads [127]. The group has also demonstrated that TGF-β1-triggered rearrangement of cytoskeleton in mesangial cells occurs by detection of smooth muscle–actin in stress fibers and it can bring about assembly redistribution of focal adhesion complex [127]. TGF-β regulated actin reorganization via ERK signalling is cell type-specific and may not be crucial for all migratory cells. However, its significance has been established in mammary epithelial cells and cortical tubule epithelial cells when blockage of ERK signalling prevented TGF-β-induced stress fiber formation [134].

TGF-β-mediated phosphatidylinositol-3-kinase (PI3K) pathway activation leads to further activation of Rac and Cdc42, which triggers effector molecules that aid migration [133]. Furthermore, TGF-β has also been associated with podosome and invadopodia formation by operating through Src kinase–Tks5 pathway to carryout proteolytic ECM degradation for cellular propulsion. In addition, Src and PI3K have been shown to partake in podosome assembly in osteoclasts and endothelial cells [135–137]. Moreover, it has been demonstrated that RhoA regulates podosome formation through PI3K activation in osteoclasts [137]. TGF-β-mediated SMAD independent cytoskeleton remodelling is outlined in Fig. 4.

## TGF-β1-mediated targeting of Aβ and Tau

Various studies conducted over the years have advocated the role of TGF-β1 in addressing the amyloid deposition in the brain. These studies have shown TGF-β1 as an essential player in promoting microglial neuroprotective functions that in turn inhibit Aβ accumulation [67]. An early study carried out by Wyss Coray and group has demonstrated the contrasting ramifications of TGF-β1 on amyloid deposits in cerebral vasculature and parenchyma of the brain. A clear correlation was observed between the increase in astroglial TGF-β1 production and an overall reduction in Aβ plaque deposition in parenchyma, hippocampus, and neocortex of hAPP mice [67]. The same study has also implicated TGF-β1 in having an amyloidogenic effect on cerebral vasculature, thus contributing to cerebrovascular amyloidosis/cerebral amyloid angiopathy (CAA) [112]. The fundamental basis for TGF-β1-induced Aβ clearance in brain parenchyma was the strong activation of microglial macrophage inflammatory protein 1α (MIP-1α), which activates microglia-dependent clearance mechanisms in its presence [67].

**Fig. 5:**
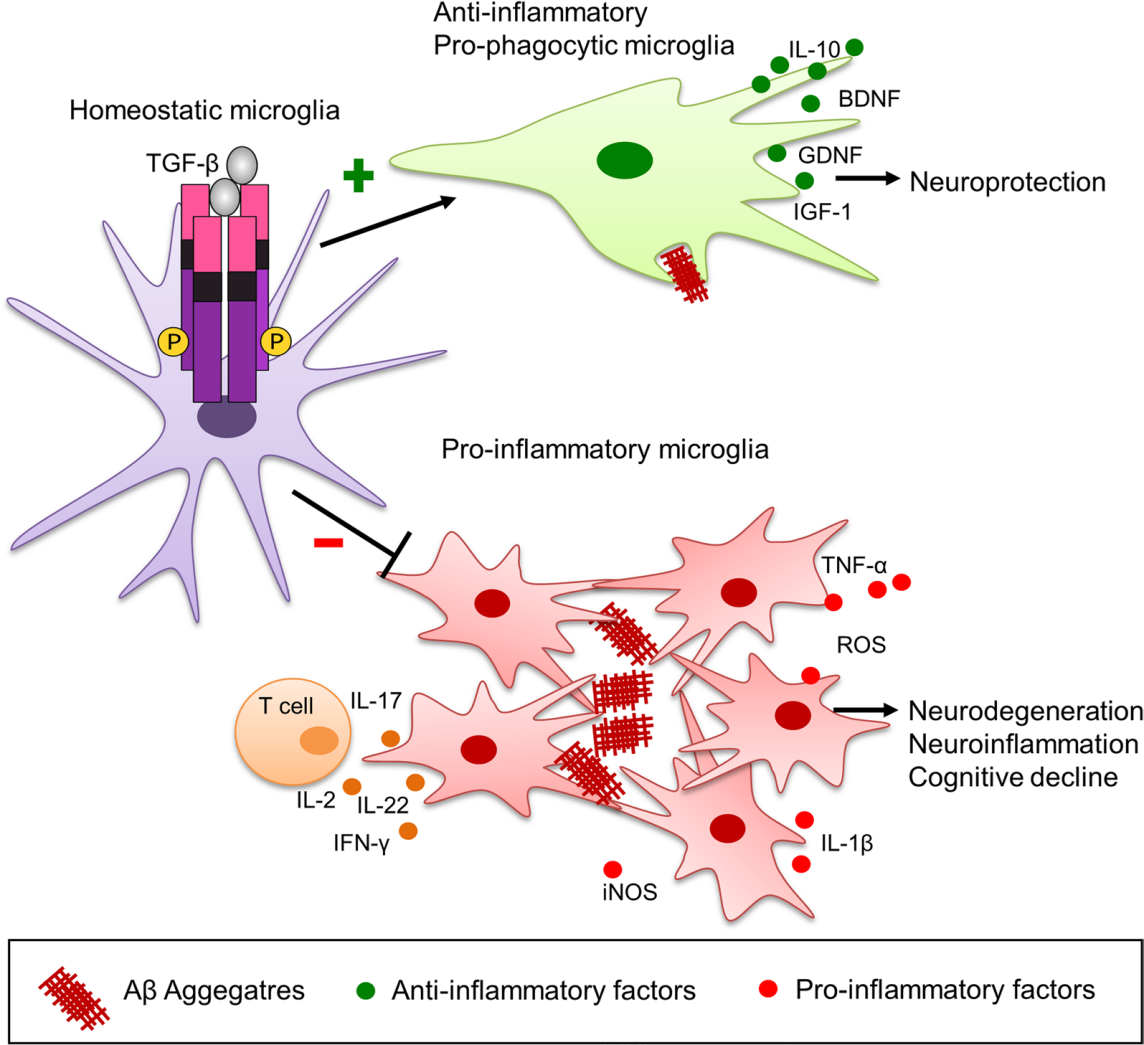
Role of TGF-β1 in targeting Aβ deposits in Alzheimer's disease. TGF-β1 has been shown to promote microglia-mediated neuroprotective action of targeting and clearing Aβ plaques through phagocytosis, while its role in blocking clustering of microglia and perpetuation of subsequent inflammation towards Aβ deposits has also been suggested. Furthermore, TGF-β1 shows an overall neuroprotective function through (i) inhibition of proinflammatory state of microglial activation and suppressing the release of cytokines facilitating inflammation, such as TNF-α, IL-1β, IL-2, IL-17, IL-22 and IFN-γ, (ii) maintenance of anti-inflammatory state by upkeeping IL10 levels, (iii) maintenance of IGF-1, BDNF, GDNF and other neurotrophic factors through modulation of their expression levels and preventing neurodegeneration and associated cognitive decline

Neuroinflammation, a major contributing factor in Alzheimer's pathogenesis, is perpetuated by glial activation-induced by amyloid/Tau deposits [138]. TGF-β has been linked to amelioration of such neurotoxicity by reducing glial activation (microglial pro-inflammatory phenotype), decreasing expression of pro-inflammatory cytokines, such as TNF-α, iNOS and IL-1β, increasing expression of trophic factors essential for neuronal survival-like IGF-1, GDNF, and BDNF, and maintaining neuroprotective anti-inflammatory cytokine IL-10 levels [139]. It has also been implicated in suppressing T-cell-mediated neuroinflammation by decreasing pro-inflammatory cytokines IFN-γ, IL-2, IL-17, and IL-22 derived from T-cells, thus establishing itself as a preventive or therapeutic agent in Aβ1–42-induced AD rat model [139]. Research conducted on a transgenic animal model with mutant human presenilin 1 and a chimeric mouse/human β-amyloid precursor protein (APP_swe_) has demonstrated that blood-derived microglia can carry out more effective phagocytosis of amyloid deposits in comparison with resident microglia in vivo as well as in-vitro. Higher levels of antigen presentation in the formers' case could be the probable reason [140, 141]. Recent studies have validated the therapeutic potential of injecting primary cultured rat microglia, bone-marrow-derived microglia (BMDM) [140], mouse bone-marrow-derived microglia-like cells (BMDML) [89, 142], and peripheral blood-derived microglia-like (PBDML) cells [143] for the elimination of amyloid deposits by a directional migration and consequent, cell-specific phagocytosis [144]. We have ideated the potential promising role of TGF-β1 in facilitating microglia-mediated Tau targeting based on the seminal paper by Kuroda et al. (2020). They have clearly delineated the role of TGF-β1 in Aβ phagocytosis through three modes: i) Conditioned medium treatment: The conditioned medium extracted from BMDML cells consisted of high levels of TGF-β1. Treatment of cultured mouse microglia with the same TGF-β1-rich medium resulted in TGF-βR1 activation on resident microglia, increased microglial Smad2/3 phosphorylation and Aβ phagocytosis, while TGFβR1 inhibitor suppressed those effects. ii) Recombinant TGF-β1 treatment: confirmatory similar results were observed when treatment with recombinant TGF-β1 was given, which were again found to be suppressed by TGF-βR1 inhibitor. iii) Intrahippocampal transplantation of BMDML cells: injecting these cells in mouse AD model increased the TGF-β1 levels and TGF-β1 receptor expression in brain resident microglia while reducing Aβ levels in the hippocampus. Treatment with inhibitor (SB525334) hampered the above-mentioned effects. The role of TGF-β1 in targeting Aβ-associated pathology is summed up in Fig. 5.

Since the extracellular localization of Tau has been established as per the prion spread hypothesis of Alzheimer’s, it has become evident that it can be targeted in a way similar to targeting of extracellular Aβ deposits. Moreover, the above mentioned results from Kuroda et al. have brought forward a direct link between TGF-β1 and microglial Aβ phagocytosis, such that we believe that this study can be extrapolated to also target extracellular Tau [89].

**Fig. 6:**
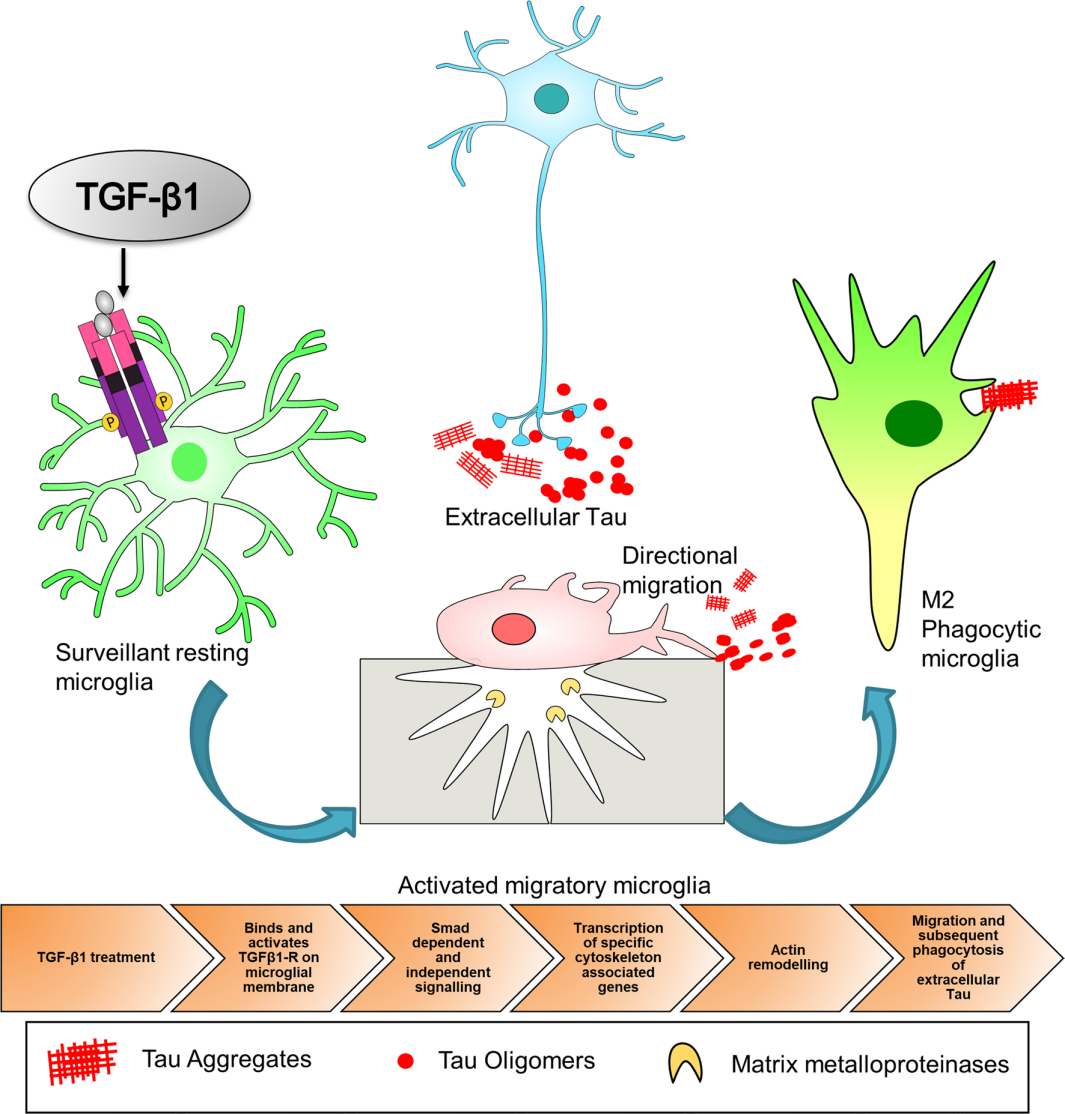
TGF-β-directed microglia-mediated targeting of extracellular Tau: A Hypothesis. Enrichment of TGF-β1 in the local milieu would lead to activation of TGF-β type II receptor which intern leads to phosphorylation, recruitment and heterodimerization of TGF-β type I receptor on microglial membrane. Further downstream signalling leads to SMAD dependent or SMAD independent pathway activation. The constituent components of both these pathways help remodulate cytoskeletal elements to facilitate directional migration towards extracellular Tau, culminating into its phagocytic clearance by microglia

Along those lines, the effect of TGF-β1 on microglial phagocytosis aimed at targeting Tau deposition should be explored. Enabling a transforming growth factor-β1 (TGF-β1)-rich local environment can direct microglia towards a migratory pro-phagocytic phenotype which we hypothesize can target extracellular seeded Tau aggregates/oligomers in a similar way as it does amyloid deposits. This is represented in Fig. 6.

## Possible challenges

Employing TGF-β1 to direct microglia to phagocytose extracellular Tau aggregates/oligomeric seeds could pose several challenges. A possible problem could be addressing the ability of TGF-β1 to clear brain parenchyma and dumping the pathoprotein deposits onto the cerebral blood vessels. This could thus contribute to elevated amyloid/Tau levels in cerebral vasculature and would eventually result into cerebral amyloid angiopathy (CAA). The study by Kuroda et al. that forms the cornerstone of our hypothesis was carried out on APdE9 mice which is not a viable model to understand TGF-β1-linked cerebral amyloid angiopathy (CAA) [89]. Therefore, more detailed analysis using a defined CAA model is needed before drawing any conclusion on the causal effect of TGF-β1 on CAA. Another significant contradiction is that TGF-β1 is a well-known perpetrator of epithelial to mesenchymal transition in cancerous cells. Therefore, its prolonged usage in AD therapeutics could possibly translate into an elevated risk of acquiring cancer. A program for its regulated and microglia-specific release needs to be thought of to address this issue. In addition, further cause for concern is the conflicting evidence from another study conducted using BV-2 microglia. This study concluded that TGF-β1 can inhibit the chemotactic migration and clustering of overactive microglia towards Aβ aggregates through SMAD-dependent down-regulation of chemokine CCL5. SB431542, a TGF-β1 inhibitor, significantly reduced TGF-β1-induced SMAD2 phosphorylation and restored the expression of CCL5, thereby mitigating the inhibitory effect of TGF-β1 on amyloid induced microglial migration and clustering [90]. Thus, further studies need to be undertaken to clearly characterise the exact nature and constituent components of TGF-β1 signalling mediating microglial migration before diving into its therapeutic usage for Alzheimer's.

## Concluding remarks and future perspective

In this review, we have focused on the probable utilization of TGF-β1 as a possible efficient strategy to approach the problem of extracellularly propagated Tau in Alzheimer’s. AD-associated dysregulation of microglia impairs its neuroprotective functions and drives it towards a pro-inflammatory phenotype that is devoid of phagocytic ability. Impaired phagocytosis in turn contributes to excessive accumulation of Aβ and Tau proteins. As per the literature, we have clearly delineated that TGF-β1 can help tune the microglia towards an anti-inflammatory phase. It has also been established that TGF-β1 has the ability to remodulate cytoskeletal components of various cells to aid migration towards a target. Its role in clearing amyloid plaque deposits via phagocytosis has also been defined. Propagation of Tau protein (monomer), Tau oligomer and Tau aggregates from one cell to another can be curbed by engulfing them just after their release and prior to their uptake by healthy cells. Thus, this review suggests that microglia can be modulated towards an anti-inflammatory, pro-phagocytic phenotype that can clear extracellular Tau seeds using TGF-β1. We are interested in addressing the prion-like seeding effect shown by Tau in AD using this directive after intercepting the challenges that this strategy brings. Further studies need to be undertaken to establish the potential applicability of this paradigm.

## Data Availability

Data sharing is not applicable to this article as no data sets were generated or analyzed during the current study.

## Abbreviations

TGF-β: Transforming Growth Factor-β
AD: Alzheimer's disease
Aβ: Amyloid-beta
NFTs: Neurofibrillary tangles
APP: Amyloid precursor protein
MAPT: Microtubule-associated protein Tau
CNS: Central nervous system
FTLD-17: Fronto-temporal dementia with Parkinsonism linked to chromosome 17
PHF: Paired helical filaments
SF: Straight filaments
AMPA: α-Amino-3-hydroxy-5-methyl-4-isoxazolepropionic acid
VAMP8: Vesicle-associated membrane protein 8
ISF: Intestinal fluid
CSF: Cerebrospinal fluid
HMW: High molecular weight
PET: Positron Emission Tomography
MRI: Magnetic resonance imaging
IFN-γ: Interferon gamma
TNF-α: Tumour Necrosis Factor alpha
IL-1β: Interleukin-1β
IL-3: Interleukin-3
ROS: Reactive oxygen species
NO: Nitric oxide
DAM: Disease-associated microglia
APOE: Apolipoprotein E
TREM2: Triggering receptor expressed on myeloid cells 2
TYROBP: TYRO protein tyrosine kinase-binding protein
CX3CL1: C–X3–C Motif Chemokine Ligand
ALK5/TβRI: Activin-like kinase 5/TGF-β type I receptor
TβRII: TGF-β type II receptor
MAPK: Mitogen-Activated Protein Kinase
NF-κB: Nuclear factor κB
PI3K: Phosphatidylinositol-3-kinase
Tmem119: Transmembraneprotein 119
P2ry12: Purinergic Receptor P2Y12
Sall1: Sal-like 1
Olfml3: Olfactomedin-like 3
Hex-b: Hexosaminidase Beta
SiglecH: Sialic acid binding Ig-like lectin H
Fcrls: Fc receptor-like S
MFGCC-E8: Milk fat globule–EGF factor 8 protein
Bcl-2: B-cell lymphoma 2
Bcl-xL: B-cell lymphoma-extra large
Wnt: Wingless-related integration site
GSK-3β: Glycogen synthase kinase 3 beta
EMT: Epithelial-to-Mesenchymal Transition
MMPs: Matrix metallopeptidases
ECM: Extracellular matrix
LAP: Latency-Associated Peptide
LTBP: Latent TGF-β-Binding Protein
HIC-5: Hydrogen peroxide inducible clone
NET1: Neuroepithelial cell transforming gene 1
α SMA: Alpha Smooth Muscle Actin
RhoA: Ras-homolog gene family, member A
Cdc42: Cell division control protein 42 homolog
ROCK: Rho-associated protein kinase
WASP: Wiskott–Aldrich Syndrome protein
WAVE: WASP-family verprolin–homologous protein
Arp2/3: Actin-related proteins 2/3
ERK: Extracellular-regulated kinase
Tks5: Tyrosine kinase substrate with 5 SH3 domains
CAA: Cerebral amyloid angiopathy
MIP-1α: Microglial macrophage inflammatory protein 1α
IGF-1: Insulin-like growth factor 1
GDNF: Glial cell-derived neurotrophic factor
BDNF: Brain cell-derived neurotrophic factor
TNF-α: Tumour Necrosis Factor alpha
IL-10: Interleukin 10
iNOS: Inducible nitric oxide synthase
BMDM: Bone-marrow-derived microglia
BMDML: Mouse bone-marrow-derived microglia-like cells
PBDML: Peripheral blood-derived microglia-like
LOAD: Late-onset Alzheimer's disease
MT1-MMP or MMP-14: Membrane-type matrix metalloproteinases
